# Unsupervised deep learning reveals prognostically relevant subtypes of glioblastoma

**DOI:** 10.1186/s12859-017-1798-2

**Published:** 2017-10-03

**Authors:** Jonathan D. Young, Chunhui Cai, Xinghua Lu

**Affiliations:** 1 Department of Biomedical Informatics, University of Pittsburgh, 5607 Baum Blvd, Pittsburgh, PA 15206 USA; 2 Intelligent Systems Program, University of Pittsburgh, 5607 Baum Blvd, Pittsburgh, PA 15206 USA; 3 Center for Causal Discovery, University of Pittsburgh, 5607 Baum Blvd, Pittsburgh, PA 15206 USA

**Keywords:** Deep learning, Unsupervised learning, Cancer, Glioblastoma multiforme, Deep belief network, Gene expression, Model selection

## Abstract

**Background:**

One approach to improving the personalized treatment of cancer is to understand the cellular signaling transduction pathways that cause cancer at the level of the individual patient. In this study, we used unsupervised deep learning to learn the hierarchical structure within cancer gene expression data. Deep learning is a group of machine learning algorithms that use multiple layers of hidden units to capture hierarchically related, alternative representations of the input data. We hypothesize that this hierarchical structure learned by deep learning will be related to the cellular signaling system.

**Results:**

Robust deep learning model selection identified a network architecture that is biologically plausible. Our model selection results indicated that the 1st hidden layer of our deep learning model should contain about 1300 hidden units to most effectively capture the covariance structure of the input data. This agrees with the estimated number of human transcription factors, which is approximately 1400. This result lends support to our hypothesis that the 1st hidden layer of a deep learning model trained on gene expression data may represent signals related to transcription factor activation. Using the 3rd hidden layer representation of each tumor as learned by our unsupervised deep learning model, we performed consensus clustering on all tumor samples*—*leading to the discovery of clusters of glioblastoma multiforme with differential survival. One of these clusters contained all of the glioblastoma samples with G-CIMP, a known methylation phenotype driven by the *IDH1* mutation and associated with favorable prognosis, suggesting that the hidden units in the 3rd hidden layer representations captured a methylation signal without explicitly using methylation data as input. We also found differentially expressed genes and well-known mutations (*NF1*, *IDH1*, *EGFR*) that were uniquely correlated with each of these clusters. Exploring these unique genes and mutations will allow us to further investigate the disease mechanisms underlying each of these clusters.

**Conclusions:**

In summary, we show that a deep learning model can be trained to represent biologically and clinically meaningful abstractions of cancer gene expression data. Understanding what additional relationships these hidden layer abstractions have with the cancer cellular signaling system could have a significant impact on the understanding and treatment of cancer.

## Background

Understanding the cellular signal transduction pathways that drive cells to become cancerous is fundamental to developing personalized cancer therapies that decrease the morbidity and mortality of cancer. Most research studying cellular signaling pathways has concentrated on a handful of signaling proteins in a hypothesis-driven framework. This study uses a deep learning approach to study cancer signaling systems in a large-scale data-driven fashion, with an overall goal of understanding the cellular signaling pathways that drive or cause cancer. Towards this goal, we used unsupervised deep learning to find meaningful structure and relationships in cancer gene expression data.

Deep learning models (DLMs) originated from artificial neural networks (ANN) and learn alternate representations of the original input data. A DLM is composed of multiple layers of latent variables (hidden nodes or units, in the ANN jargon) [1–5], which learn to represent the complex statistical structures embedded in the data, such that different hidden layers capture statistical structure of different degrees of complexity. In other words, DLMs learn novel representations of the statistical structure of the input data through hierarchical decomposition. For example, if one trains a convolutional neural network (a type of DLM) with three hidden layers on a dataset of face images (in order to learn how to recognize specific people in images), the units in these three layers capture abstract representations at different levels. The model may use the 1st hidden layer units (the layer that is closest to input data) of the trained network to capture edges of different orientations present in the original image [6, 7]. The 2nd hidden layer units may learn representations of different parts of a face [7] (e.g., nose, mouth, eye, ear), by combining edges of different orientations (represented by the units in the 1st hidden layer). Finally, units in the 3rd hidden layer may represent generic faces [4, 7], which can be thought of, or represented as, combinations of parts of a face. In this way, deep learning finds hierarchical structure in data by finding alternate representations (often of lower dimension) that best encode the information in the original image. Once a DLM is trained, it can be used to detect a specific person in an image or, depending on the type of model, it can be used to generate new face images that mimic the distribution of the input images. In this study, we aim to use DLMs to find the hidden layer representations of cancer gene expression data that likely represent the state of the signaling systems in cancer cells.

**Fig. 1:**
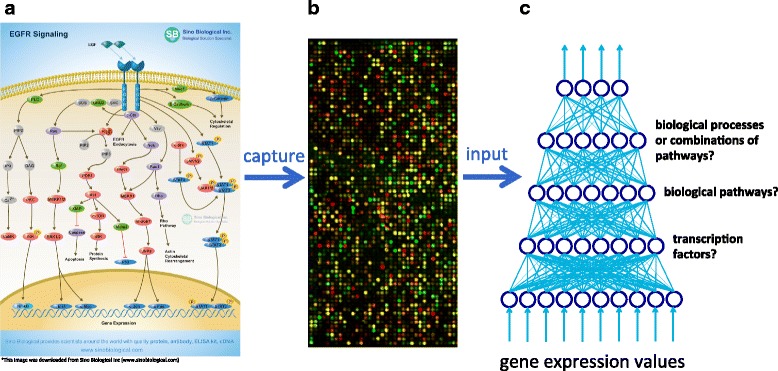
Inferring the activation states of signaling pathways with deep learning. (**a**) Example EGFR signaling cascade. (**b**) A microarray representing gene expression values. (**c**) Representation of a DBN (fine-tuning encoder network only) trained on gene expression values. Hypothesized biological entities whose activation states may be represented by units in the 1st, 2nd, and 3rd hidden layers are displayed on the *right*

More specifically, we hypothesize that the activation states of signaling pathways regulating transcriptomic activities in cancer cells can be learned using DLMs (Fig. 1). Cancer is a multi-process disease, in that a cancer cell usually has multiple aberrant pathways—each pathway consists of a set of hierarchically organized signaling molecules (Fig. 1a)—driving differentially expressed genes (DEGs) that are involved in multiple oncogenic processes. A transcriptomic profile of a tumor is a convoluted mixture of expressed genes regulated by active pathways in tumor cells, but the information related to the hierarchical organization of pathways is “collapsed” and distinct signals from different pathways become inseparable (Fig. 1b). Discovering aberrant signals in cancer cells requires de-convolution (decomposition) of such signals, and DLMs are particularly well suited for such a task due to their ability to perform hierarchical decomposition. For example, the hierarchical structure of a signaling pathway (Fig. 1a) could be simulated by the hierarchical structure in a DLM trained on gene expression data (Fig. 1c). Since transcription factor (TF) activation dictates the finest covariance structure of gene expression, which is at the bottom of the signaling pathway in Fig. 1a, the 1sthidden layer in our DLM (Fig. 1c) may capture the signals encoded by TFs. And just as there are different pathways being activated that regulate transcription factor activation in the middle of Fig. 1a, the 2nd hidden layer of our DLM may represent the activation states of different biological pathways. Continuing with this analogy, units in the 3rd hidden layer could represent biological processes (e.g., inflammation or metastasis) or combinations of pathways. In this way, we aim to learn the hierarchical representation of the signals underlying cancer gene expression data with deep learning.

Recently, we demonstrated that DLMs could be used to learn and represent the cellular signaling system [8, 9]. In one study, we showed that a DLM, more specifically a multi-modal deep belief network (DBN) and a semi-restricted multi-modal DBN can learn representations of the cellular signaling system shared by rat and human cells [9]. In the same study, we also demonstrated that a trans-species DLM could accurately predict human cell responses to a set of unknown stimuli based on data from rat cells treated with the same stimuli. In a more recent study, we showed that DLMs can learn representations of the yeast transcriptomic system, and that the hidden units of these DLMs can be mapped to real biological entities, such as transcription factors and well-known yeast signaling pathways [8]. Another group, Liang et al., integrated multiple types of genomic cancer data with a multi-modal DLM trained to regenerate the observed data (the entire omics profile), but their goal was not to infer aberrant signals, i.e., the differences in signaling between normal and cancer cells [10], as we did in this study.

In this study, we investigated the optimal architectures of DBN-based models to learn accurate representations of the signals underlying cancer transcriptomic changes across 17 cancer types. We show that a DLM can provide novel abstract representations, enabling us to reveal molecular subtypes of cancers, e.g., subtypes of glioblastoma multiforme (GBM), that exhibit significant differences in outcomes. Our analysis revealed different potential disease mechanisms (major driver genes) underlying these molecular subtypes.

## Methods

### Data

The data used in this study were obtained from The Cancer Genome Atlas (TCGA) Data Portal [11], and included transcriptomic data for 17 different cancer types and non-cancer organ-specific tissue controls, all downloaded from the TCGA Data Portal (Table 1). The total size of the dataset was 7528 samples by 15,404 genes.

**Table 1:** Number of samples for each cancer type in our dataset

Tissue Type	Number of Samples
Bladder urothelial carcinoma (BLCA)	403
Breast invasive carcinoma (BRCA)	1073
Esophageal carcinoma (ESCA)	183
Colon adenocarcinoma (COAD)	283
Glioblastoma multiforme (GBM)	481
Head and neck squamous cell carcinoma (HNSC)	508
Kidney renal clear cell carcinoma (KIRC)	525
Kidney renal papillary cell carcinoma (KIRP)	288
Liver hepatocellular carcinoma (LIHC)	364
Lung adenocarcinoma (LUAD)	509
Lung squamous cell carcinoma (LUSC)	498
Ovarian serous cystadenocarcinoma (OV)	559
Prostate adenocarcinoma (PRAD)	491
Rectum adenocarcinoma (READ)	93
Stomach adenocarcinoma (STAD)	236
Thyroid carcinoma (THCA)	499
Uterine corpus endometrial carcinoma (UCEC)	535
Total	7528

We discretized the expression value of a gene in a tumor to 1 or 0 based on whether or not the expression value significantly deviated from the expression observed in normal tissue. To achieve this, we fit the expression values of each gene in each cancer type to a Gaussian distribution based on the non-cancer control samples only from the same tissue of origin. We then set the expression status of a gene in a tumor to 1 (aberrant) if it was outside the 0.001 percentile of distribution of control samples (on either side), otherwise we set it to 0. For genes with low expression variance in normal cells, i.e., standard deviation of expression smaller than 0.2, we used 3-fold change to determine whether the genes were differentially expressed in tumor cells. Through this discretization process, we identified genes that were potentially relevant to the cancer process (aberrantly expressed in cancer only) rather than just using the whole gene expression profile of a cell, which includes both physiological and pathological signals. The gene expression changes due to copy number alteration were also masked; as such changes are not regulated by the cellular signaling system, but are due to genomic alterations. In each tumor, we identified the genes that had expression changes and the genes that had copy number alterations, i.e., GISTIC2.0 [12] score equal to +1 (amplification) and GISTIC2.0 score equal to −1 (deletion). When we discovered gene expression up-regulation co-occurring with a corresponding copy number amplification, or a gene expression down-regulation co-occurring with a corresponding copy number deletion, we masked the gene expression change—as this co-occurrence suggested that the expression changes were caused by the DNA copy number alteration.

### Preprocessing

Feature selection was performed to remove genes with low Bernoulli variance because of their general lack of information. We created datasets with different numbers of features by using different variance thresholds for feature selection. We identified genes that had an expression status of 1 (or 0) in 90% (Bernoulli success probability) of tumors and removed them from further analysis due to their low variance. This resulted in a dataset with 7160 features. We repeated this process using a Bernoulli success probability of 0.95 to create a dataset with 9476 features. We also removed any genes that were highly correlated with a specific cancer type or tissue type, by removing all genes with a Pearson correlation coefficient, with respect to cancer or tissue type labels, greater than 0.85.

### Model specification

**Fig. 2:**
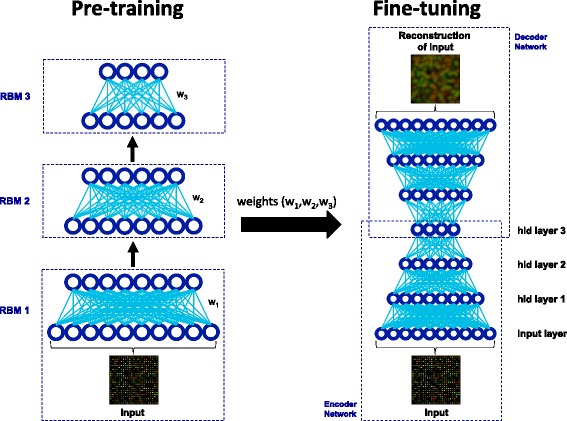
Deep belief network (DBN) with two phases: pre-training and fine-tuning. Each RBM in the pre-training phase iteratively learns lower dimensional representations of the input (gene expression microarray) one RBM at a time. These lower dimensional representations are then used as input to the next RBM. The pre-training phase learns a set of weights (*W* = *w*_*1*_, *w*_*2*_, and *w*_*3*_) that is used to initialize the fine-tuning phase. The fine-tuning phase optimizes the weights by minimizing the cross-entropy error

The specific deep learning strategy used for this study is called a DBN (layer-by-layer pre-training followed by “up-down” fine-tuning) [1, 13–15]. Although it may be clearer and more explicit to refer to the strategy used in this study as a stacked restricted Boltzmann machines–deep autoencoder (SRBM–DA), we will use the more traditional DBN terminology [1, 13–15] for the sake of being consistent with the literature. Learning of a DBN consists of two major phases: a pre-training phase and a fine-tuning phase (Fig. 2).

In the pre-training phase, the hierarchical DBN model is treated as multiple restricted Boltzmann machines (RBMs) stacked on top of each other, such that the top layer of an RBM is used as the bottom layer of another RBM above it. Learning of the parameters (often referred to as weights *W*) of the pre-training phase, starts with the learning of the weights of each of the stacked RBMs in a layer-by-layer fashion.

In more detail, the pre-training phase is a deep generative model consisting of a stack of RBMs. An RBM is a 2-layered undirected probabilistic network that seeks to learn a latent representation (often of lower dimension) of the input data by optimizing the instantiation of the latent variables (latent representation) and the weights of the network in order to best allow the latent representation to regenerate the input data [14]. The objective of an RBM is to find the parameters *θ* (including a weight matrix *W* and offset vectors (biases) *b, c*) that maximize the probability of the input data (visible layer, *v*) [1].

1 $$ \underset{\theta }{\mathrm{argmax}}P(v)={\sum}_hP\left(v,h\right) $$

2 $$ P\left(v,h\right)=\frac{1}{Z}{e}^{-E\left(v,h\right)} $$

3 $$ E\left(v,h\right)=-{h}^T Wv-{c}^Tv-{b}^Th $$

The joint probability *P*(*v*, *h*) of the hidden *h* and visible *v* layers is a function of the energy function *E*(*.*) [1]. *Z* is a normalization factor or partition function.

In the fine-tuning phase, the DBN model is “unfolded” (leading to a network often referred to as a deep autoencoder, DA) in order to learn to reconstruct the input data using the weights learned during the pre-training phase. The fine-tuning phase performs a global optimization of all weights using stochastic gradient descent and the backpropagation algorithm, with the goal of minimizing the difference between the distribution of the data and the distribution formed by the model’s reconstructions of the data (cross-entropy error) [13, 14]. Deep networks can be somewhat difficult to train [16, 17]. Using the weights learned during pre-training to initialize a DA, as opposed to random initialization, seems to improve the generalization of the completely trained DBN by minimizing the variance of all possible solutions to the DBN [13, 18].

In more detail, a DA is a multi-layered network composed of an encoder and decoder network [14]. The encoder network learns multiple encodings (hidden layer representations) of the input by propagating the input forward through the network (as one would in a neural network using a linear transformation and a nonlinearity/squashing function), learning alternate representations of the input at each hidden layer [1, 14]. Once the final hidden layer is computed, the decoder network propagates in reverse [1, 14]. When propagating in reverse, the DA uses the final hidden layer of the network to attempt to regenerate the original input data. The output of fine-tuning (DA) is a reconstruction of the input data based on decoder propagation through the network. Cross-entropy error can be used to determine how close these reconstructions are to the original input, and the weights can be updated in the appropriate direction (trained using backpropagation of error derivatives and stochastic gradient descent) [1, 14] in order to minimize the cross-entropy error. Reconstruction error (mean squared error between the data and the reconstructions of the data) is often used to monitor learning progress. More detailed descriptions of training DBNs can be found in [8, 14, 15].

We implemented a DBN [14] using the Python programming language and the Theano library (a symbolic numerical computation python library) [19]. This implementation is compatible with Mac OS or Linux computing environments and is capable of utilizing GPUs if available.

### Model selection

In order to investigate the impact of the hyperparameters (network architecture, learning rate, training duration) on modeling the cancer transcriptomic data, we performed a series of model selection experiments. Model selection was performed using a modified 8-fold cross-validation. In order to speed up our model selection (allowing us to explore more sets of hyperparameters), while still training on a large percentage of our dataset (considering our dataset had a somewhat small number of samples relative to the number of features), we only performed four folds of an 8-fold split of the data. Our strategies for deep learning model selection were guided by articles from Bengio [20] and Hinton [21]. A combined random and grid search approach [20] were used with a goal of finding the set of hyperparameters that minimized the average test set reconstruction error and prevented overfitting, while also significantly reducing the dimensionality of the data (i.e., final (top) hidden layer with around 100 units). Please see the Results and discussion section for more information on model selection.

### Consensus clustering

After model selection, we trained the deep learning model and then computed and collected the top hidden layer (the most abstract) representations for each sample. We performed consensus clustering on the top hidden layer representations (i.e., 3rd hidden layer representations, meaning the final 100–200 dimensional projections of the input data) of each tumor as calculated by our trained DBN models; as well as, on the high-dimensional input data alone. Consensus clustering was performed using the ‘ConsensusClusterPlus’ [22] package from the R statistical programming language [23], using agglomerative hierarchical clustering with Euclidean distance and average linkage. Consensus clustering performs multiple trials (in this case 100) of clustering based on randomly sampling a subset of the data (in this case 80%). Each sample is given a final cluster assignment at the end of each trial. A consensus matrix is created after all trials have completed. A consensus value is a number between 1 and 0 that represents how often two samples did or did not cluster together, respectively.

The output of the consensus clustering was a dendrogram and an associated consensus matrix heatmap (dimensions = number of samples by number of samples), representing how often samples clustered together in repeated clustering trials. The DBN models with the lowest percentage of ambiguous clustering (PAC) [24] values and most visually informative heatmaps were selected for further analysis. The PAC represents the proportion of data points in the consensus matrix with intermediate (between 0.8 and 0.2) consensus values, meaning that the two samples clustered together in some runs of clustering, but not in others.

### Kaplan-Meier survival analysis

A Kaplan-Meier plot was created using the clustering assignments from the consensus of consensus clustering for GBM samples. Kaplan-Meier plots were created using the ‘survival’ package in the R statistical computing language [23]. *P*-values were calculated using the log-rank test.

### Correlation between genes and clusters

Correlation studies were performed to find the differentially expressed genes or mutations that correlated with each GBM cluster. The Pearson correlation between each differentially expressed gene (input features) and GBM cluster was measured using the ‘cor’ function in R. The Pearson correlation between each mutation (TCGA GBM somatic mutation data version “2015-01-27”) and GBM cluster was also measured using the ‘cor’ function in R. Functions of example genes were obtained from www.genecards.org and the Gene Ontology Consortium (http://geneontology.org/).

## Results and discussion

### Model selection

This study concentrated on finding the network architecture of a DBN model that was capable of learning “optimal” representations of cancer expression data, which is a model selection task. We performed a series of model selection experiments to find the best set of hyperparameters (e.g., number of hidden layers, number of units in each hidden layer, learning rates, training epochs, batch size, etc.). Approximately 1500 different sets of hyperparameters, including models with up to five hidden layers, were evaluated by cross-validation, representing approximately 1.5 months of computation time on a single Tesla k40 GPU.

**Fig. 3:**
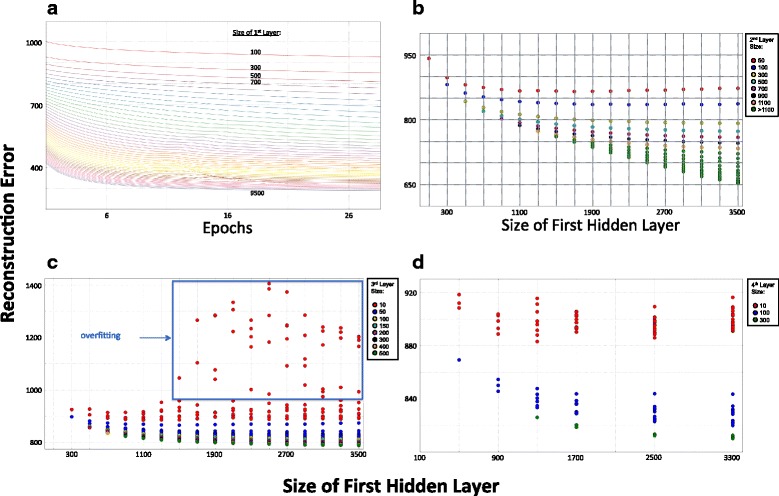
Model selection cross-validation reconstruction error. For all plots in this figure, the y-axes represent reconstruction error. (**a**) Reconstruction error for multiple sizes of a one hidden layer network. The size of the 1st hidden layer increases by 200 units for each consecutive curve. (**b**) Reconstruction error for two hidden layer networks. (**c**) Reconstruction error for three hidden layer networks. The *blue rectangle* represents network architectures that overfit the data. (**d**) Reconstruction error for four hidden layer networks

We started model selection by performing a random search over all hyperparameters, in which hyperparameters were randomly sampled from a range of values. We performed this search subject to some constraints, such as decreasing hidden layer size (e.g., hidden layer 1 always larger than hidden layer 2, etc.) and pre-defined maximum unit thresholds for hidden layer sizes. Based on these results, we selected a partial set of hyperparameters (including pre-training and fine-tuning learning rates, batch size, and pre-training and fine-tuning epochs of training duration) that appeared to perform well over a broad range of hidden layer architectures. Using this partial set of hyperparameters, we performed an extensive grid search over hidden layer architectures (from 1 to 5 hidden layers with varying number of hidden units in each layer) and evaluated the resulting reconstruction errors (Fig. 3). For all experiments in Fig. 3, only the number of hidden layers and number of units in each hidden layer varied. The other hyperparameters (i.e., learning rates, number of pre-training and training epochs, and batch size) were fixed.

Figure 3a shows how reconstruction error changes with respect to the size of the 1st hidden layer and training epochs for networks with only a *single hidden layer*, i.e., a single RBM that is then fine-tuned. As expected, the reconstruction error for a single hidden layer network decreases as the size of the hidden layer increases, and does not provide much insight into choosing the size of the 1st hidden layer. A single hidden layer DBN cannot learn the hierarchical relationships that we are interested in discovering. Therefore, we explored DBNs with multiple hidden layers.

Figure 3b shows the model selection results for different two hidden layer architectures, where the number of hidden units in the 1st hidden layer range from 100 to 3500 (across x-axis), and the number of units in the 2nd hidden layer range from 50 to 3300 (indicated by color code). Each circle in this graph (and Fig. 3c, d) represents the reconstruction error for a network architecture. Figure 3b shows that, with a small number of hidden units (50 – 100 units) in the top (2nd) hidden layer, increasing the size of the 1st hidden layer beyond 1100 does not lead to a significant reduction in reconstruction error. Indeed, as the size of the 1st hidden layer increases beyond 1100 units, the reconstruction errors remain flat and show a tendency to increase slightly. In contrast, when the size of 2nd (top) hidden layer is relatively large (> 500), the reconstruction errors continue to decrease as the size of the 1st hidden layer increases.

We hypothesized that, since a DBN is an encoding machine, an optimal model should be able to encode the input data and pass it through an information bottleneck—a model with a very small number of hidden units in the top layer of the network. Such a model would require each of the layers below this bottleneck to capture as much information as possible in order to efficiently pass the bottleneck (i.e., maintaining a low reconstruction error). As such, one can search for an optimal architecture by starting with a very small number of hidden units in the top layer and selecting the optimal number of hidden units layer-by-layer starting from the 1st hidden layer (closest to input data).

Figure 3c shows how reconstruction error changes as the sizes of the 1st, 2nd, and 3rd hidden layers change (units in 2nd hidden layer ranged from 100 to 2300). What really stands out in this graph is that overfitting is observed when the 1st hidden layer is greater than 1300 and the number of hidden units in the top hidden layer is set to 10 (overfitting indicated by a blue rectangle in the figure). Figure 3d shows how reconstruction error for four-hidden-layer DBNs changes as the sizes of the 1st, 2nd, 3rd, and 4th hidden layers change (2nd hidden layer ranged from 100 to 2100 and 3rd hidden layer from 50 to 500). Similar to Fig. 3c, d also shows overfitting when the top hidden layer size is set to 10 and the 1st hidden layer is large. We also examined five-hidden-layer networks, which showed results similar to Fig. 3d (results not shown).

In total, these results suggest that the DBN begins to capture noise in the data when the 1st hidden layer size is greater than 1300 units. Accordingly, a 1st hidden layer size around 1300 units should provide the optimal encoding of the data when the number of hidden units in the top hidden layer (the information bottleneck) is small. We hypothesized that the 1st hidden layer captures the signals encoded by TFs in human cells, and our results suggest that 1300 hidden units most effectively captures the covariance structure (hypothesized to be signals of TFs) within the data at the level of abstraction of the 1st hidden layer. Interestingly, our hypothesis agrees surprisingly well with the current consensus on the number of human TFs (~1400) estimated through analyzing the human genome [25]. These results also correlate with Chen et al. [8], who found nearly a one-to-one mapping between hidden units in the first hidden layer and yeast transcription factors.

As previously mentioned, we searched for optimal hidden layer sizes by finding ‘elbows’ in the plot of reconstruction error vs. hidden layer size, where the reconstruction error decreases less rapidly (as can be seen in Fig. 3b, c, and d). We then set the top hidden layer size to be 100 – 200 units to provide a relatively rich representation, while avoiding unnecessary complexity. We found that DBNs with four hidden layers (Fig. 3d) or 5-hidden layer networks (not shown) didn’t offer much, if any, improvement over a 3-layer network. We selected four 3-hidden-layer network architectures ([1100-500-100], [1300-700-100], [1300-700-150], [1400-1000-200]) with different combinations of hidden units in the “optimal” range. Next, we performed a random search over the learning rates for the four network architectures selected above and evaluated the reconstruction errors. Finally, we decided to use six different sets of hyperparameters (including network architecture) to test their ability to capture statistical structures in cancer gene expression data (Table 2).

**Table 2:** Model selection results. Six different hyperparameter sets for final training of DBN

Set ID	Hidden Layer Sizes			Learning Rates		Epochs		Input Size
	1st	2nd	3rd	pretrain	train	pretrain	train	
1	1100	500	100	7.75E-05	2.41E-03	14	101	9476
2	1300	700	100	7.75E-05	2.41E-03	14	91	9476
3	1300	700	150	7.75E-05	2.41E-03	14	88	9476
4	1300	700	150	7.75E-05	2.41E-03	14	62	9476
5	1400	1000	200	3.03E-03	3.26E-03	14	40	7160
6	1300	700	150	7.75E-05	2.41E-03	14	97	7160

### Clustering tumors based on DBN-derived representations

**Fig. 4:**
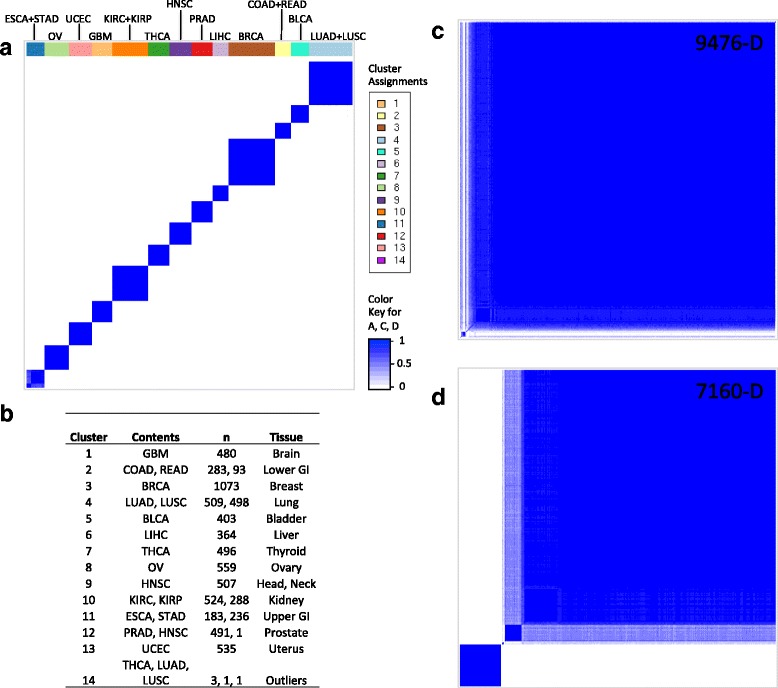
Consensus clustering of all samples. (**a**) Consensus clustering of the 3rd hidden layer representations from our DBN model (model 5 in Table 2) captured tissue-specific clustering. The cancer types within each cluster are shown on *top*. (**b**) Composition of each of the clusters learned in part a, including the number of samples of each cancer type in each cluster. (**c**) Consensus clustering results when simply using the high-dimensional (9476 features/genes) raw data without any dimensionality reduction. (**d**) Consensus clustering results for high-dimensional (7160 features/genes) raw data without any dimensionality reduction. All heatmaps evaluated at k = 14 (number of clusters). *Dark blue* corresponds to samples that always cluster together (consensus value = 1). *White* corresponds to samples that never cluster together (consensus value = 0).* Lighter shades of blue* are intermediate between 1 and 0

The purpose of training a DBN model with a large number of tumors of multiple cancer types was to use a large amount of data to enhance our learning of statistical structures—to potentially reveal different disease mechanisms. Based on the assumption that the learned representations reflect the state of cellular signaling systems, it would be interesting to learn if these representations can be used to reveal cancer subtypes that share a common pattern of pathway perturbation. To this end, we represented each tumor using the states of the hidden units in the top (3rd) layer and performed consensus clustering. Figure 4 shows that the 3rd hidden layer representations from a trained DBN (Fig. 4a) clustered drastically better than the high-dimension raw gene expression profiles (Fig. 4c, d). When tumors were represented by the 9476 input gene features, consensus clustering failed to find any meaningful clusters (i.e., multiple clusters consisting of a large number of samples).

While it is tempting to use a clustering approach to find common cancer subtypes across multiple cancer types, this approach is complicated by the fact that certain pathways exhibit tissue-specific expression, and clustering will be dominated by these tissue-specific features. This will eventually lead to the clustering of all tumor samples according to tissue type, as demonstrated in the study by Hoadley et al. [26]. Indeed, we also found that virtually all of our tumor samples clustered according to tissue type (Fig. 4a). For example, the top right cluster in the heatmap in Fig. 4a (cluster 4, colored light blue) consisted of all lung tissue samples (lung adenocarcinoma and lung squamous cell carcinoma) except for two outliers.

This tissue-specific clustering occurred despite our best attempts to remove all tissue-specific genes by representing a tumor using only genes with extremely high variance (Bernoulli success probability, 0.70) to train DBNs, and then perform consensus clustering (results not shown). Using these high variance genes reduced our number of features (genes) to 2674, with each of these genes being differentially expressed in more than 2250 tumor samples. The genes in this 2674 set cannot be tissue-specific because most cancer/tissue types in our data set have only ~500 tumor samples or less (except breast cancer and lung tissue, which have ~1000 tumors). These results indicate that there are tissue-specific pathway perturbation patterns that lead to a tissue-specific covariance structure of DEGs, which were captured by the DBN, and in turn recognized by consensus clustering. These results illustrate the limitations of using gene-expression-related features (e.g., gene expression, copy number alteration, and DNA methylation) to study disease mechanisms shared by tumors across different tissue types [26]. Therefore, different approaches to studying disease mechanisms should be explored.

### Within tissue type clustering revealed clinically relevant subtypes

Although it is difficult to search for common disease mechanisms across multiple cancer types due to the aforementioned limitations, we hypothesized that, within a given tissue type, clustering using DBN-learned representations may reveal distinct disease mechanisms. Since the survival data for glioblastoma multiforme (GBM) patients from TCGA was relatively more complete than other cancer types in our data set (allowing us to perform more robust survival analysis), we studied GBM in more detail. Previously, Verhaak et al. [27] selected a set of genes as features and performed clustering of GBM tumors from TCGA based on their expression values. However, manually selecting features may introduce bias, and therefore we set out to investigate if an unbiased representation of the data would lead to different results. We first used the raw input gene expression data as features and performed consensus clustering, but failed to find any clusters (data not shown). These results underscore the motivation for the feature-selection approach adopted by Verhaak et al. [27].

**Fig. 5:**
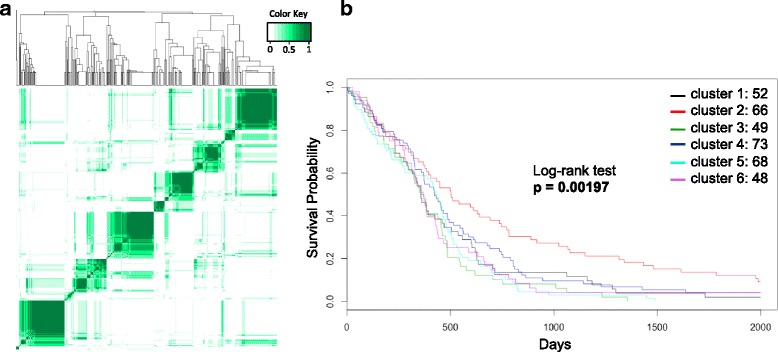
GBM consensus clustering ensemble results and corresponding Kaplan-Meier plot. (**a**) Heatmap and dendrogram for GBM consensus of consensus clustering results for six major clusters (k = 11). Dark green corresponds to samples that always clustered together (consensus value = 1). *White* corresponds to samples that never clustered together (consensus value = 0). *Lighter shades of green* are intermediate between 1 and 0. (**b**) Kaplan-Meier plot for six largest clusters in part a. Each GBM cluster is represented by a *colored curve* in the plot. *Top right* of figure shows the number of samples in each cluster

We then set out to investigate whether an unbiased representation learned by a DBN would reveal subtypes (clusters) of GBM. We trained six DBN models with different architectures and hyperparameters (see Table 2), performed consensus clustering using the results from each model (top layer representations), and we pooled the results to build a ‘consensus of consensus clustering’. The heatmap in Fig. 5a shows the general agreement across GBM cluster assignments as derived from the six DBN models. This is a type of ensemble learning where each of the six models gets to ‘vote’ for which samples should be clustered together. Using PAC scores (see Methods) as a selection criterion, we identified six major clusters (Fig. 5a). We explored this six major cluster separation further to see if the learned clusters were clinically relevant.

Figure 5b shows the Kaplan-Meier plot for the samples in the six major GBM clusters (Fig. 5a). There is a difference in survival between the patient clusters and in particular the red curve/cluster seems to have better survival or prognosis relative to the other clusters. The *p*-value for Kaplan-Meier plot using the log-rank test was *p* = 0.00197.

**Fig. 6:**
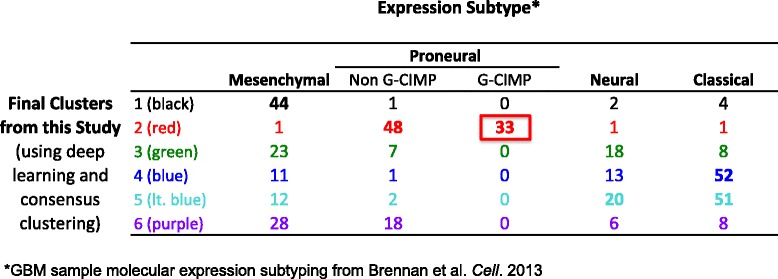
GBM subtypes in each cluster from Fig. 5b based on molecular subtyping from Brennan et al. [28]

GBM is a highly aggressive malignant brain tumor. Previous molecular analysis of GBM tissue samples by Verhaak et al. identified four molecular subtypes: mesenchymal, proneural, neural, and classical [27]. The analysis of the four subtypes identified by Verhaak et al. did not reveal significant differences in survival between the four clusters, but the tumors did exhibit different responses to treatments. More recently, Brennan et al. further divided tumors within the proneural subtype into G-C island methylation phenotype (G-CIMP) and non G-CIMP subtypes [28] based on DNA methylation data. Here, our DBN-derived representations separated GBM tumors into six clusters (Fig. 5a), and our subtyping revealed significant differences in patient survival (Fig. 5b), indicating that our novel representations provide more information than using individual gene expression values as features. We compared our subtyping (learned using deep learning and consensus clustering) with the known subtyping of our TCGA GBM samples (Fig. 6) as published by Brennan et al. [28].

Figure 6 shows the contents of our six GBM clusters based on the subtype published by Brennan et al. [28]. Most of our GBM clusters included tumor samples belonging to multiple different known subtypes. Exceptions to this were our black and red clusters (see Fig. 5b for cluster colors). The black cluster consisted of mostly mesenchymal subtype. The red cluster (cluster with best prognosis) in the Kaplan-Meier plot consisted of almost entirely proneural subtype samples. Interestingly, this red cluster captured all of the samples with the G-CIMP phenotype and the majority of the non G-CIMP proneural tumors, but assigned the rest of non G-CIMP tumors to the purple cluster. The G-CIMP phenotype (samples with hypermethylation at CpG islands) subgroup of GBM has been shown in previous studies to have better survival [28–30]. These results indicate that without utilizing DNA methylation data, DBN learned representations accurately captured the impact of DNA methylation on expression—an indication that our novel representations may reflect disease mechanisms at the pathway level.

### Novel clusters provide information regarding disease mechanisms

**Fig. 7:**
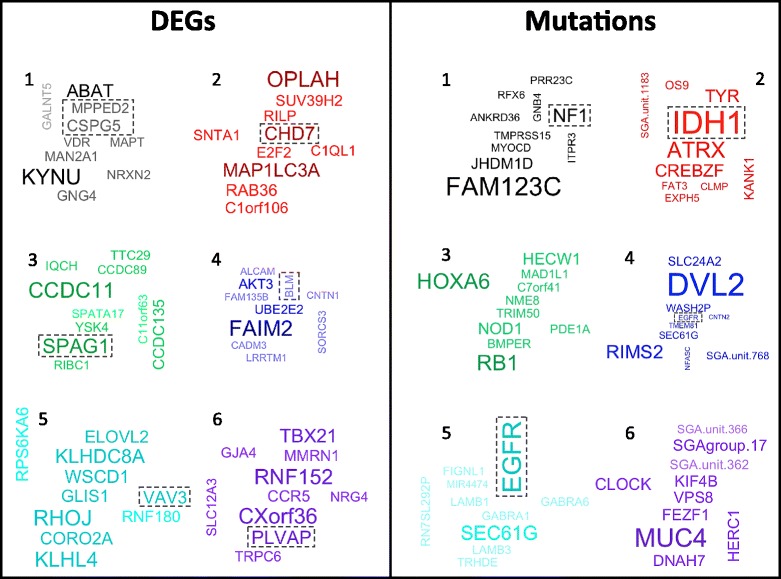
Word clouds for the top 10 DEGs or mutations with the largest correlations with each GBM cluster. Each GBM cluster is represented by a word cloud of genes, colored according to the corresponding curve in the Kaplan-Meier plot (Fig. 5b). The size and color of each gene in each word cloud correspond to the relative strength of the correlation for that gene. The largest and darkest words in the word clouds correspond to the strongest correlations. Each cluster’s word cloud was created independently. Therefore, the differential sizes of each gene (representing correlations) is only relevant when compared to other genes in that cluster. Gene sizes should not be compared across different clusters

We investigated the six GBM clusters further using correlation analysis to find DEGs and mutations that were associated with each cluster. Figure 7 (left panel) shows word clouds for the top 10 DEGs with the largest positive correlations with each GBM cluster. Each word cloud of genes is colored according to their corresponding cluster color in the Kaplan-Meier plot. For example, the red colored genes represent the DEGs or mutations with the largest correlations with the red cluster (cluster with the best prognosis). We found genes in each of these groups with functions relevant to cancer. For example, *CHD7* is highly correlated with the red cluster and is involved in DNA packaging and chromatin binding. *CSPG5* and *MPPED2* (highly correlated with the black cluster) are involved in nervous system development. *BLM* (blue cluster) is involved in DNA binding and regulation of cell proliferation. *VAV3* (light blue cluster) is involved in GTPase activator activity. *SPAG1* (green cluster) is known to be involved in epilepsy and pancreatic cancer. *PLVAP* (purple cluster) may function in microvascular permeability.

Figure 7 (right panel) shows word clouds for the top 10 mutations with the largest positive correlations with each GBM cluster. This correlation analysis yielded many well-known mutations involved in cancer and GBM. *IDH1* is the mutation with the strongest correlation with the red cluster, which includes all tumors belonging to G-CIMP subtype of GBM [28]. This finding is biologically sensible in that it is known that mutations in *IDH1* lead to significant changes in DNA methylation, which underlie the G-CIMP. Similarly, *NF1* mutations are strongly associated with the black cluster (corresponding to the mesenchymal subtype) and are known to be frequently mutated in the mesenchymal subtype [27].

The above results reveal connections between genomic alterations and DEGs specifically associated with each subtype, which provide information about the disease mechanisms for each subtype. It is reasonable to assume that the genomic alterations associated with a cluster likely perturb pathways underlying the subtype, and hidden units in our DBN models could potentially represent the states of these pathways. Any aberration in these pathways causes a change in the expression of the DEGs associate with that cluster. Studying the potential causal relationships between mutation events and the changing states of hidden units may provide information about how mutations affect signaling pathways in cancer cells.

## Conclusions

In this study, we showed that an unsupervised DBN can be used to find meaningful low-dimensional representations of cancer gene expression data. More specifically, first, we designed a rigorous model selection scheme, which enabled us to determine the optimal number of hidden units in the 1st and 3rd hidden layers of our model. We hypothesized that the 1st hidden layer likely represented the TFs utilized by cancer cells and our results correlate with current knowledge of the number of TFs. Second, we showed that consensus hierarchical clustering of GBM tumors using the unbiased representations (the top (final) hidden layer units) revealed more robust clustering results than clustering based on the raw gene expression data. Third, we showed that clinically relevant information was encoded in the representations learned by our DBN. This was demonstrated through the discovery of a subtyping of GBM with differential prognosis, which previously was not discovered by TCGA. Our methods identified a subtype of GBM enriched with the G-CIMP phenotype without using DNA methylation data, and our analysis can partially attribute this subtype to the mutation of *IDH1*. This also agrees with current knowledge. Further investigation may reveal disease mechanisms underlying the different GBM clusters. What role do these genes/mutations have in GBM? What role do they play in survival?

This study represents a novel application of the deep learning algorithm developed by Hinton and Salakhutdinov [14] in the cancer bioinformatics domain. To our knowledge, unsupervised deep learning has not been used to find hidden structure within cancer gene expression data for the purposes of providing insight into disease mechanisms of tumors and patient survival. As for the possible future enhancement of the model, we conjecture that a sparse version of our DBN may more readily encode cellular pathways. A trained model needs to be able to represent all cancer pathways in order to fit the data from the thousands of tumors studied here, however a given tumor likely only hosts a small number of aberrant pathways. A sparse DLM can limit the number of active hidden units in a given layer representing a tumor, thus it theoretically could perform better. This will be investigated in future studies.

## Abbreviations

ANN: Artificial neural network
DA: Deep autoencoder
DBN: Deep belief network
DEG: Differentially expressed gene
DLM: Deep learning model
GBM: Glioblastoma multiforme
G-CIMP: G-C island methylation phenotype
PAC: Percentage of ambiguous clustering
RBM: Restricted Boltzmann machine
SRBM**–**DA: Stacked restricted Boltzmann machines–deep autoencoder
TCGA: The cancer genome atlas
TF: Transcription factor
